# Safety and tolerability of high doses of taspoglutide, a once-weekly human GLP-1 analogue, in diabetic patients treated with metformin: a randomized double-blind placebo-controlled study^1^,^2^

**DOI:** 10.1111/j.1464-5491.2010.02990.x

**Published:** 2010-05

**Authors:** R Ratner, M Nauck, C Kapitza, V Asnaghi, M Boldrin, R Balena

**Affiliations:** Medstar Research InstituteHyattsville, MD, USA; * Diabeteszentrum, Bad Lauterberg im HarzNeuss, Germany; † Profil Institut für Stoffwechselforschung GmbHNeuss, Germany; ‡ F. Hoffmann-La Roche LtdBasel, Switzerland; § Roche, NutleyNJ, USA

**Keywords:** clinical studies, drug treatment, glucagon-like peptide 1, new drugs, Type 2 diabetes

## Abstract

**Aims:**

The study objective was to investigate the safety and tolerability of up-titration to high doses of taspoglutide, a once-weekly human glucagon-like peptide-1 analogue, in subjects with Type 2 diabetes inadequately controlled on metformin alone.

**Methods:**

In this double-blind phase II trial, subjects were randomized to placebo or taspoglutide (20 mg; three separate groups) administered once weekly by subcutaneous injection for 4 weeks. This was followed by dose maintenance at 20 mg, or titration to 30 mg (20/30) or 40 mg (20/40) once weekly with matched placebo for an additional 4 weeks. Subjects were monitored for adverse events (AEs) throughout the study and 4-week follow-up.

**Results:**

One hundred and twenty-nine subjects were randomized and treated [mean age 57 years, mean baseline glycated haemoglobin (HbA_1c_), 7.9%]. The most frequently reported AEs were nausea and vomiting. The number of patients reporting gastrointestinal AEs did not increase following titration to higher doses of taspoglutide or when continuing the initial 20 mg regimen. Three subjects were withdrawn from the study as a result of gastrointestinal AEs (one before and two after titration to higher doses). Although not designed to investigate efficacy, improvement in glycaemic control was observed in all active arms of the study. The proportion of subjects achieving HbA_1c_ < 7.0% after 8 weeks of treatment was 72, 53 and 70% in the 20/20-, 20/30- and 20/40-mg arms, respectively, vs. 19% for placebo.

**Conclusions:**

Taspoglutide was safe, well tolerated at high doses and efficacious for lowering HbA_1c_. Up-titration of dose was not associated with a worsening AE profile.

## Introduction

Taspoglutide is an analogue of the human incretin hormone glucagon-like peptide 1 (GLP-1) that is currently in phase III clinical development for treatment of Type 2 diabetes mellitus (Type 2 DM). Taspoglutide is designed to make therapeutic use of the glucoregulatory properties of GLP-1, which enhances glucose-dependent insulin secretion, suppresses inappropriately elevated glucagon secretion, slows gastric emptying, and reduces food intake [1–3]. Taspoglutide contains aminoisobutyric acid substitutions at positions 8 and 35 of the native GLP-1 peptide. These modifications confer a 12-fold increase in stability over GLP-1 when incubated in rat serum and render the compound resistant to dipeptidyl peptidase-4 (DPP-4) mediated inactivation [4]. Taspoglutide shows high binding affinity to the human GLP-1 receptor and assessment of its relative activity on cyclic adenosine monophosphate stimulation in cells expressing the GLP-1 receptor suggests that it is fully active [4]. Taspoglutide improved glucose tolerance, normalized postprandial glucose, improved glycaemic control and insulin sensitivity, and reduced excess body weight in a rat model of Type 2 DM [5]. Its resistance to proteolytic degradation and a zinc-based sustained-release formulation confer an extended half-life and allow for once-weekly subcutaneous administration (s.c.). An early clinical trial showed that a single administration of taspoglutide 8 or 30 mg to patients with Type 2 DM reduced fasting and postprandial plasma glucose levels, stimulated insulin secretion and reduced glucagon levels [6]. Moreover, reductions in glycated haemoglobin (HbA_1c_), fructosamine and body weight were observed in a dose-finding phase II study [7]. In the latter study, mixed meal tests before and after treatment in a subset of patients revealed that taspoglutide significantly improved pancreatic B-cell function, as measured by improvements in insulin secretion rates and B-cell glucose sensitivity [8].

The primary aim of the current study was to investigate the safety and tolerability of up-titration to high doses of taspoglutide in patients with Type 2 DM and to determine whether dose titration could be used to administer doses higher than the anticipated therapeutic doses while limiting the expected GI side effects.

## Subjects and methods

### Study design

**FIGURE 1:**
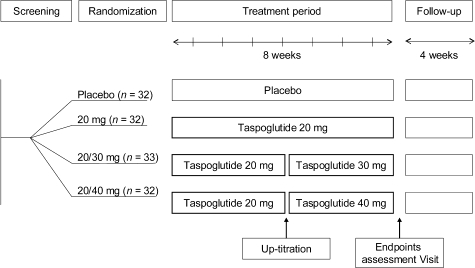
Study design.

This study was a randomized, double-blind, placebo-controlled phase II trial conducted at 27 sites in six countries (Australia, France, Germany, Mexico, Peru and the USA) between April 2007 and October 2007. The study consisted of a double-blind 8-week treatment period with an additional 4-week follow-up. It was planned to enrol approximately 120 subjects into the study, assigned randomly and equally to each of the treatment groups. This sample size was determined by practical considerations rather than formal calculations. Subjects on stable metformin monotherapy were randomized to receive placebo s.c. once weekly for 8 weeks, or 20 mg taspoglutide s.c. once weekly for 4 weeks, followed by 4 weeks of 20 mg once weekly (20/20), or titration up to 30 mg once-weekly (20/30) or 40 mg once-weekly (20/40) taspoglutide (Fig. 1). The doses and titration scheme were selected using modelling and simulation. The predicted drug exposure was adequately tolerated in patients with Type 2 DM uncontrolled by metformin in a previous trial where taspoglutide was infused at doses up to 800 μg/day (F. Hoffmann-La Roche; data on file).

A common clinical trial protocol was approved for each site by an institutional review board. All subjects provided written informed consent before participation and the study was conducted in accordance with the principles described in the Declaration of Helsinki, including all amendments through the 1996 South Africa revision (World Medical Association Declaration of Helsinki) [9].

Randomization was implemented via a central system using a stratified randomization procedure based on disease severity (HbA_1c_ < 8.0% or ≥ 8.0%) to avoid imbalances between treatment groups.

The primary endpoint of this study was GI tolerability, which was assessed by comparing the number of subjects who withdrew from the study because of GI adverse events (AEs) in the active treatment groups with those in the placebo group. Secondary endpoints included changes in fasting plasma glucose, HbA_1c_ and body weight, and pharmacokinetic parameters.

### Study population

The study population comprised men and post-menopausal or surgically sterilized women aged 18–75 years with Type 2 DM, treated with a stable daily dose of metformin monotherapy for at least 3 months before screening. The dosage of metformin was not adjusted during the study. Key inclusion criteria at screening were HbA_1c_ between 7.0% and 9.5% (inclusive), fasting plasma glucose > 7.0 mmol/l and ≤ 13.3 mmol/l, body mass index (BMI) > 25.0 kg/m^2^ and ≤ 45.0 kg/m^2^, and weight less than or equal to ±10% for at least 3 months before screening.

Subjects with serious co-morbidities or abnormalities in laboratory tests were excluded from the study, as were those who had previously been treated with GLP-1 receptor agonists (including GLP-1 itself) at any time, or with other glucose-lowering medications (apart from metformin) or weight-loss medications within 12 or 6 weeks, respectively. The use of other glucose-lowering medications, glucose-lowering herbal remedies or weight-loss medications during the study was not permitted (i.e. required withdrawal from blinded study drug). ACE, thiazide diuretics, thyroid hormones and/or lipid-lowering medications were permitted but only with dose(s) stable for at least 6 weeks prior to screening.

### Study procedures

Eligible subjects who had given prior written informed consent attended clinic visits weekly during the 8-week treatment period, beginning on the day of first treatment (day 0/week 0), and during the 4-week follow-up period. At each visit, vital signs and body weight were monitored, AEs were recorded and fasting blood samples were taken. A 12-lead electrocardiogram was conducted at weeks 0, 4, 8 and 11. Study drug was administered before breakfast during the clinic visits in weeks 0–7. Subjects were asked to follow their pre-study diet and exercise plan and metformin regimen throughout the study. Subjects reported to the study site for weekly visits. At each visit the lyophilized peptide was reconstituted at the study site with the diluent (zinc chloride solution). Subjects in the placebo group received 0.9% sodium chloride (NaCl). Study medication was administered by personnel not involved with the preparation of study drug to maintain the blinding and injected s.c. in the abdomen in a different site each time, according to a given scheme.

AEs were categorized as: (i) ‘mild’ if transient with discomfort, but no disruption of normal daily activity; (ii) ‘moderate’ if sufficient discomfort to reduce or affect daily activity; or (iii) ‘severe’ if the discomfort rendered the subject unable to work or perform normal daily activity. A withdrawal rule for GI AEs was implemented: ‘if severe nausea and/or vomiting requiring therapy persist up to 24 h before the next scheduled administration of the drug, the subject should be withdrawn from the study.’

Standard laboratory assessments (haematology and biochemistry) were conducted weekly. Fasting plasma glucose was measured weekly and HbA_1c_ was measured at weeks 0, 4, and 8. Population pharmacokinetics were assessed using sparse sampling. Central laboratories (Covance: Sydney, Australia; Singapore; Geneva, Switzerland; Harrogate, UK; Indianapolis, IN, USA) were used to measure all laboratory parameters including drug concentrations of taspoglutide; the latter were measured using a liquid chromatography/tandem mass spectrometry assay with a lower limit of quantification of 7.5 pmol/l.

### Statistical analysis

Safety data were summarized for all subjects who received at least one dose of randomized study drug (the safety population). AEs were categorized according to preferred terms in the Medical Dictionary for Regulatory Activities (MedDRA), version 10.1 (Maintenance and Support Services Organization; Chantilly, VA, USA). Descriptive statistics were used to report the safety results. Data on secondary endpoints were analysed for the intent-to-treat (ITT) population (all subjects who were randomized to the study, received study drug and had a valid baseline and at least one post-baseline HbA_1c_ measurement), using the last observation carried forward for dropouts. Data were evaluated using analysis of covariance (ancova) to determine the least squares (LS) means of change from baseline. The ancova model included treatment as the fixed effect term and baseline as the covariate.

## Results

### Subject demographics and disposition

One hundred and thirty-three subjects were randomized. Four subjects (one from each of the treatment groups) did not receive any study drug; therefore, the safety population consisted of 129 subjects (Fig. 1). The ITT population comprised 125 subjects. The safety population was well balanced across the treatment groups in terms of key demographic characteristics (Table 1). During the study, many subjects received common medications for cardiovascular risk factors; most frequently, statins (22%), ACE inhibitors (21%) and fibrates (5%). No subjects reported using glucose-lowering medications other than metformin and taspoglutide.

**Table 1:** Baseline characteristics (safety population, *n* = 129)

		Taspoglutide dose		
	Placebo once weekly *n* = 32	20 mg once weekly *n* = 32	20/30 mg once weekly *n* = 33	20/40 mg once weekly *n* = 32
Male/female, *n* (%)	13/19 (41/59)	15/17 (47/53)	15/18 (45/55)	13/19 (41/59)
Age (years)	56 ± 2	57 ± 2	55 ± 2	60 ± 2
Weight (kg)	92.9 ± 3.5	89.8 ± 3.8	88.3 ± 3.0	90.2 ± 3.9
BMI (kg/m^2^)	33.2 ± 1.0	33.3 ± 0.9	31.6 ± 1.0	31.5 ± 0.9
Duration of diabetes (years)	7 ± 1	6 ± 1	8 ± 1	7 ± 1
HbA_1c_ (%)	7.8 ± 0.1	8.0 ± 0.1	8.0 ± 0.1	7.8 ± 0.1
Fasting glucose (mmol/l)	9.4 ± 0.3	9.4 ± 0.3	8.9 ± 0.3	8.9 ± 0.3

Data are mean ± standard error.

BMI, body mass index; HbA_1c_, glycated haemoglobin.

### Safety and tolerability

No subject was withdrawn from the study because he/she met the criterion for withdrawal as a result of GI AEs as defined in the study protocol; however, three out of 129 subjects were withdrawn because of GI AEs at the request of the investigator. Two of these subjects were in the 20/30-mg arm (dyspepsia, vomiting) and one was in the 20/40-mg arm (upper abdominal pain). Overall, 16 subjects withdrew prematurely from the study; one from the placebo arm, three from the 20/20-mg arm and six each from the 20/30- and 20/40-mg arms. Seven of these subjects left the study because of AEs, including the three GI AEs described above; one from the placebo arm (cardiac arrhythmia), two from the 20/30-mg arm (the cases of dyspepsia, vomiting described above) and four from the 20/40-mg arm (the case of upper abdominal pain described above, and cases of ventricular extrasystoles, contusion or hypoglycaemia).

**FIGURE 2:**
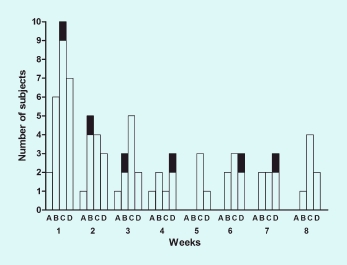
The number of subjects with nausea; mild/moderate (white) or severe (black) over the 8-week study period in the: A, placebo; B, 20/20 mg; C, 20/30 mg; and D, 20/40 mg once-weekly taspoglutide arms (safety population).

The most commonly reported AEs were GI signs and symptoms (Table 2). Nausea was most prevalent after the first and second weekly administrations, decreasing with subsequent injections (Fig. 2). Of the subjects who reported nausea, most reported it as mild to moderate in severity and in most cases the nausea resolved spontaneously. A similar, temporal relationship of vomiting in terms of duration and severity was also reported (data not shown). Overall, the number of subjects who reported GI AEs decreased over time and did not increase following titration to the higher doses (30 or 40 mg). The number of subjects who reported GI AEs was 16 (48%) before titration vs. 12 (41%) after titration in the 20/30-mg arm, and 12 (38%) before titration vs. 10 (36%) after titration in the 20/40-mg arm. While a reduction in the number of subjects reporting GI AEs was observed in all groups between the first and second 4-week treatment periods, the greatest reduction was seen in subjects who remained on the 20-mg dose of taspoglutide throughout the 8-week study period, with a decrease of approximately 48% [from 17 (53%) to 9 (30%)].

**Table 2:** Most frequently reported adverse events (safety population, *n* = 129)*

	Number (%) of subjects			
		Taspoglutide dose		
Adverse event	Placebo once weekly *n* = 32	20 mg once weekly *n* = 32	20/30 mg once weekly *n* = 33	20/40 mg once weekly *n* = 32
Nausea	4 (13)	12 (38)	17 (52)	11 (34)
Headache	4 (13)	5 (16)	2 (6)	3 (9)
Diarrhoea	3 (9)	4 (13)	7 (21)	3 (9)
Fatigue	1 (3)	3 (9)	4 (12)	1 (3)
Vomiting	0	4 (13)	9 (27)	4 (13)
Dyspepsia	0	6 (19)	5 (15)	5 (16)
Abdominal distension	0	3 (9)	4 (12)	1 (3)

* Adverse events that began during study treatment and occurred in ≥ 10% of subjects in any treatment group.

Two serious AEs were reported in the study; cardiac arrhythmia in a subject given placebo and the suspicion of recurrence of prostate cancer in a subject in the 20/40 taspoglutide arm. Both events were considered by the investigators to be unrelated to the study drug. No safety issues with regard to vital signs, electrocardiogram or laboratory analysis were identified during this trial. Injection site reactions were observed in 13%, 69%, 52% and 59% of subjects in the placebo, 20/20-, 20/30- and 20/40-mg groups, respectively; these were considered mild or moderate, with no severe reactions reported. Most of these reactions were indurations that appeared soon after treatment administration and subsequently resolved spontaneously. These local reactions were not usually accompanied by any signs or symptoms of acute response; in many cases, the reactions had not been noticed by the subjects but were reported by the investigators who had been instructed to actively check every injection site at each visit.

Seven subjects developed signs and symptoms of hypo-glycaemia during the study: one in the placebo arm, one in the 20/20 arm, three in the 20/30 arm and two in the 20/40 arm of the study. All subjects had recorded their blood glucose values at the time of the event and only one of these events was accompanied by blood glucose concentrations ≤ 2.8 mmol/l (value = 2.8 mmol/l in a patient allocated to the 20/30-mg arm). The blood glucose values recorded at the time of the episodes in other five subjects ranged from 3.3 to 5.7 mmol/l. No severe episodes of hypoglycaemia were reported.

### Pharmacokinetics and pharmacodynamics

**FIGURE 3:**
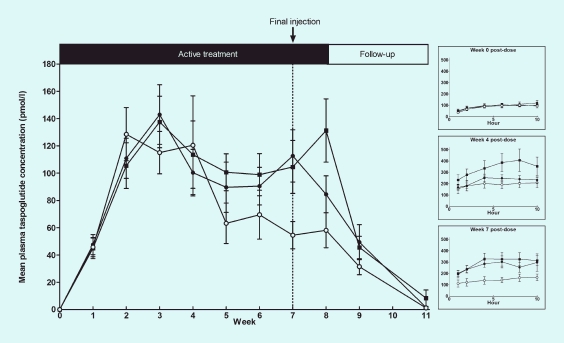
Plasma concentration (mean ± standard error) of taspoglutide in groups receiving subcutaneous administration of 20/20 mg once weekly (white circles), 20/30 mg once weekly (black circles) or 20/40 mg once weekly (black squares). All groups received 20 mg once weekly until week 3. The first doses of 30 or 40 mg once weekly were administered at week 4. Pre-dose (trough) concentrations are shown for weeks 0–7; the insets show post-dose concentrations for the first 10 h after treatment at weeks 0, 4, and 7.

Exposure to taspoglutide appeared dose proportional and time dependent (Fig. 3) and after 8 weeks of treatment median trough plasma concentration values were 44.4, 84.2 and 109.7 pmol/l at 20/20, 20/30 and 20/40 mg once weekly, respectively.

**FIGURE 4:**
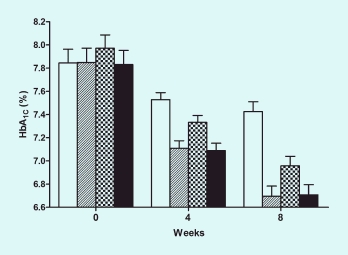
Glycated haemoglobin (HbA_1c_) (%) (last observation carried forward) over time. Placebo (white); 20/20 mg taspoglutide (diagonal shade); 20/30 mg taspoglutide (checkered); 20/40 mg taspoglutide (black). Intent-to-treat population; least squares mean + standard error.

**FIGURE 5:**
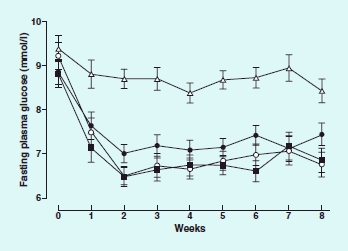
Fasting plasma glucose (FPG) concentrations change from baseline (last observation carried forward) over time. Placebo (white triangles); 20/20 mg taspoglutide (black squares); 20/30 mg taspoglutide (white circles); 20/40 mg taspoglutide (black circles). Intent-to-treat population; least squares mean *±* standard error.

Reductions in HbA_1c_ were observed in all taspoglutide-treated arms of the study (Fig. 4). At week 8, the changes from baseline in HbA_1c_ were −1.2 ± 0.1, −0.9 ± 0.1 and −1.2 ± 0.1% in the 20/20-, 20/30- and 20/40-mg arms, respectively [LS mean ± standard error (se)], vs. −0.5 ± 0.1% with placebo (*P* < 0.0001 in all groups vs. the placebo group). Moreover, the percentage of subjects achieving HbA_1c_ < 7.0% was 72% (20/20-mg group), 53% (20/30 mg) and 70% (20/40 mg) in the taspoglutide arms vs. 19% in the placebo arm. Additionally, the percentage of subjects achieving HbA_1c_ < 6.5% was 41% (20/20 mg), 21% (20/30 mg) and 37% (20/40 mg) in the taspoglutide arms vs. none in the placebo arm. Fasting plasma glucose was reduced (Fig. 5), with LS mean changes from baseline to week 8 of –2.3 ± 0.3 mmol/l (20/20, *P* < 0.0001 vs. placebo), −1.6 ± 0.3 mmol/l (20/30, *P* = 0.007 vs. placebo) and −2.2 ± 0.3 mmol/l (20/40, *P* < 0.0001 vs. placebo) in the active study arms. By comparison over the same time period, in subjects randomized to receive placebo, fasting plasma glucose levels fell by 0.6 ± 0.3 mmol/l.

Body weight decreased progressively in all groups throughout the 8-week treatment period. Changes from baseline (LS mean ± se) were −2.1 ± 0.4 kg in the 20/20 group (*P* = 0.77 vs. placebo), −3.0 ± 0.3 kg in the 20/30 group (*P* = 0.03 vs. placebo) and −2.7 ± 0.4 kg in the 20/40 group (*P* = 0.17 vs. placebo) compared with −2.0 ± 0.3 kg in the placebo group.

## Discussion

The main objective of this phase II study was to assess the safety and tolerability of doses of taspoglutide up to 30 and 40 mg once weekly, when administered following 20 mg once weekly for 4 weeks to subjects treated with stable doses of metformin monotherapy. The major finding was that titration to higher doses of taspoglutide following 4 weeks of 20 mg once weekly was not associated with a deterioration of GI tolerability.

As expected from a GLP-1 receptor agonist, early human data documented that transient nausea and vomiting were the most commonly reported AEs following administration of taspoglutide [6]. However, previous studies have demonstrated for native GLP-1 and for other drugs in this class that gradual dose escalation may help to mitigate the recognized GI side effects through induction of drug tolerance [10]. In this study, titration to 30 or 40 mg doses of taspoglutide did not result in an increase in the number of subjects reporting GI AEs. The greatest reduction in the number of GI AEs was seen in those subjects who remained on a dose of 20 mg taspoglutide throughout the study. This finding is consistent with studies of other GLP-1 receptor agonists, and with the expectation that the risk of nausea and vomiting decreases with continued drug exposure [11,12]. The pharmacokinetic analyses indicated that subjects who titrated to higher doses (30 or 40 mg of taspoglutide) had proportionally higher plasma levels of drug than those remaining on 20 mg once weekly. Significantly, no subjects withdrew from the study because of nausea. The overall low occurrence of withdrawals because of GI AEs, and the timing of these withdrawals, supports the study hypothesis that titration with taspoglutide is feasible well beyond 20 mg.

While this study was not specifically powered to assess efficacy, taspoglutide 20 mg once weekly led to a statistically significant 1.2% reduction in HbA_1c_ after 8 weeks of treatment. Furthermore, this dose enabled 72% of subjects to attain the current American Diabetes Association and European Association for the Study of Diabetes target HbA_1c_ of < 7.0% [13] from a baseline of 7.8–8.0%, with no significant hypoglycaemic episodes. Interpretation of the glycaemic data is limited by the fact that the total treatment period was only 8 weeks, and that, as planned per protocol, the majority of subjects received the highest dose of their assigned regimen for only 4 weeks, an insufficient duration of treatment to assess maximal HbA_1c_ reduction. Nonetheless, these findings suggest rapid glycaemic efficacy in terms of reduction of both HbA_1c_ and fasting plasma glucose.

It is recognized that treatment with some commonly available diabetes drugs is associated with weight gain [14], a distinct disadvantage in a patient population in whom controlling body weight is important to ameliorate the risk of diabetes complications. Despite the short treatment duration of the current study, there was a reduction in body weight in all groups studied, including placebo, with an apparent trend towards greater weight loss in the 20/30- and 20/40-mg groups. This is in line with previous observations of another human GLP-1 analogue that higher doses than necessary for glucose control still further reduce body weight, pointing to a rightward-shift in the dose–response relationship for appetite control [15].

The development of GLP-1 receptor agonists has been an important advance in the field of diabetes medicine [16]. These new therapies offer multiple improvements over existing drugs with an emphasis on providing optimal glycaemic control; however, currently approved GLP-1 receptor agonists are administered daily or twice daily. Clearly, there is still a requirement for compounds that are efficacious and safe, but also convenient to administer. Taspoglutide was developed with these optimal attributes in mind.

In summary, this study demonstrates that taspoglutide, a human once-weekly GLP-1 analogue, provides rapid glycaemic improvement with a favourable safety and tolerability profile. Although not yet studied in comparative clinical trials, the GI side-effect profile observed in this study appears to be similar to other GLP-1 receptor agonists. Additionally, this study confirms that dose titration and/or maintenance on the 20-mg dose of taspoglutide was not associated with an increased incidence of GI AEs. Although the short duration of the trial does not allow definitive conclusions, the results support the hypothesis that maximal efficacy is achieved with a 20-mg dose. Indeed up-titration of taspoglutide to 30 or 40 mg, although safe, was devoid of any additional therapeutic effect. Further studies are warranted and have commenced with the 20-mg once-weekly dose of taspoglutide to gain a better understanding of the long-term efficacy and tolerability profile.

## Abbreviations

ACE: Angiotensin-converting enzyme inhibitors
AE: adverse event
ancova: analysis of covariance
GI: gastrointestinal
GLP-1: glucagon-like peptide
HbA_1c_: glycated haemoglobin
ITT: intent-to-treat
LS: least squares
s.c.: subcutaneously
Type 2 DM: Type 2 diabetes mellitus

## References

[1] Kreymann B, Williams G, Ghatei MA, Bloom SR (1987). Glucagon-like peptide-1 7-36: a physiological incretin in man. Lancet.

[2] Holst JJ (1999). Glucagon-like peptide-1, a gastrointestinal hormone with a pharmaceutical potential. Curr Med Chem.

[3] Drucker DJ (2005). Biologic actions and therapeutic potential of the proglucagon-derived peptides. Nat Clin Pract Endocrinol Metab.

[4] Sebokova E, Christ AD, Wang HY, Sewing S, Dong JZ, Taylor J (2010). Taspoglutide: an analog of human glucagon-like peptide-1 with enhanced stability and in vivo potency. Endocrinology.

[5] Sebokova E, Benardeau A, Sprecher U, Sewing S, Tobalina L, Migliorini C (2010). Taspoglutide, a novel human once-weekly analogue of glucagon-like peptide-1, improves glucose homeostasis and body weight in the ZDF rat. Diabetes Obes Metab.

[6] Kapitza C, Heise T, Birman P, Jallet K, Ramis J, Balena R (2009). Pharmacokinetic and pharmacodynamic properties of taspoglutide, a once-weekly, human GLP-1 analogue, after single-dose administration in patients with Type 2 diabetes. Diabet Med.

[7] Nauck MA, Ratner RE, Kapitza C, Berria R, Boldrin M, Balena R (2009). Treatment with the human once-weekly glucagon-like peptide-1 analog taspoglutide in combination with metformin improves glycemic control and lowers body weight in patients with type 2 diabetes inadequately controlled with metformin alone: a double-blind placebo-controlled study. Diabetes Care.

[8] Berria R, Gastaldelli A, Nauck M, Boldrin M, Asnaghi V, Balena R (2008). Eight weeks’ treatment with R1583, a novel long-acting, human GLP-1 analogue, improves beta-cell function in metformin-treated diabetic subjects: a double-blind, placebo-controlled phase 2 study.

[9] World Medical Association Declaration of Helsinki (1997). Recommendations guiding physicians in biomedical research involving human subjects. J Am Med Assoc.

[10] Fineman MS, Shen LZ, Taylor K, Kim DD, Baron AD (2004). Effectiveness of progressive dose-escalation of exenatide (exendin-4) in reducing dose-limiting side effects in subjects with type 2 diabetes. Diabetes Metab Res Rev.

[11] Nielsen LL, Baron AD (2003). Pharmacology of exenatide (synthetic exendin-4) for the treatment of type 2 diabetes. Curr Opin Investig Drugs.

[12] Nauck MA, Meier JJ (2005). Glucagon-like peptide 1 and its derivatives in the treatment of diabetes. Regul Pept.

[13] Nathan DM, Buse JB, Davidson MB, Ferrannini E, Holman RR, Sherwin R (2009). Medical management of hyperglycemia in type 2 diabetes: a consensus algorithm for the initiation and adjustment of therapy: a consensus statement of the American Diabetes Association and the European Association for the Study of Diabetes. Diabetes Care.

[14] Purnell JQ, Weyer C (2003). Weight effect of current and experimental drugs for diabetes mellitus: from promotion to alleviation of obesity. Treat Endocrinol.

[15] Astrup A, Rossner S, Van Gaal L, Rissanen A, Niskanen L, Al Hakim M (2009). Effects of liraglutide in the treatment of obesity: a randomised, double-blind, placebo-controlled study. Lancet.

[16] Drucker DJ, Nauck MA (2006). The incretin system: glucagon-like peptide-1 receptor agonists and dipeptidyl peptidase-4 inhibitors in type 2 diabetes. Lancet.

